# Dietary Habits and Nutritional Status of Youths Living in Rural and Semi-Urban Albania in the Ongoing Nutrition Transition: Preliminary Results

**DOI:** 10.3390/children12010098

**Published:** 2025-01-16

**Authors:** Ruden Cakoni, Stefania Moramarco, Argjend Kosiqi, Angela Andreoli, Ersilia Buonomo

**Affiliations:** 1 Faculty of Medicine, Catholic University of Our Lady of Good Counsel, 1026 Tirana, Albania; ruden.cakoni@students.uniroma2.eu (R.C.);; 2 PhD School of Nursing and Public Health, University of Rome Tor Vergata, 00133 Rome, Italy; 3 Department of Biomedicine and Prevention, University of Rome Tor Vergata, 00133 Rome, Italy; 4 Department of Systems Medicine, University of Rome Tor Vergata, 00133 Rome, Italy

**Keywords:** school-aged children, overweight, obesity, dietary habits, mediterranean diet, youth nutrition, KIDMED

## Abstract

Background: Albania is undergoing a demographic, epidemiological, and nutrition transition leading to an increased prevalence of overweight and obesity among new generations. Comprehensive studies on the nutritional status and dietary patterns of youths in the country are still lacking. Methods: A cross-sectional study was conducted on a convenience sample of students (10–18 years) attending secondary schools in rural and semi-urban areas (October–November 2024). Information collected included socio-demographic data, anthropometric measurements (weight, height), and adherence to the Mediterranean Diet (MD) (KIDMED). Factors influencing dietary patterns were investigated, with a multivariate logistic regression performed to identify key drivers for poor MD adherence (AOR 95% CI). Results: In total, 426 children (47.2% females) were interviewed. Over 20% of the sample was overweight or obese, with the prevalence of these diseases decreasing with age regardless of gender. The KIDMED score highlighted suboptimal MD adherence (4.6 ± 2.5 SD), with significant differences between females and males (4.1 ± 2.4 SD vs. 5.1 ± 2.4 SD, *p* < 0.001), especially in rural areas (3.9 ± 2.4 SD vs. 4.9 ± 2.5 SD, *p* = 0.003). Dietary quality tended to decline with age. Female gender was the strongest predictor of poor MD adherence (AOR 2.08 CI: 1.34–3.22; *p* = 0.001). Conclusions: The MD is a cornerstone for ensuring the Albanian population’s long-term health and well-being. This study holds significant public health relevance in a country with high mortality rates due to cardiovascular diseases. Future nutrition interventions focused on the poor MD adherence of new generations should take into consideration geographic, cultural, and social dimensions, including gender equality.

## 1. Introduction

Over the past three decades, Albania has undergone a demographic transition that has led to a significant change in the epidemiological profile of its population, with a marked shift towards non-communicable diseases (NCDs), particularly cardiovascular diseases (CVDs) [1]. According to the most recent data from the Global Burden of Disease (GBD), approximately 93% of all deaths in Albania in 2019 were attributed to NCDs, with CVD mortality estimated at 474 deaths per 100,000 people, representing 57% of the overall mortality [2]. This health transformation has coincided with a dietary shift commonly called “nutrition transition”, characterized by eating patterns associated with a rise in metabolic disorders and diet-related NCDs of significant public health concern [3]. For example, in 2019, 24.5% of adult women and 24.9% of adult men (aged 18 years and older) were classified as obese, while diabetes was estimated to affect 7.8% of adult women and 8.9% of adult men in Albania [4].

This nutritional transition in Albania has also led to an increased prevalence of overweight and obesity among children [3]. Childhood overweight and obesity are critical public health issues, as they significantly compromise future health and development. The World Health Organization (WHO) launched the Childhood Obesity Surveillance Initiative (COSI) as a systematic process to monitor excess body weight in primary school children in the European Region. Albania joined the WHO/COSI for the first time during the 2012/2013 school year and again in 2015/2016 [5]. Data from WHO/COSI indicated that 25% of schoolchildren aged 6 to 9 years in Albania were overweight and 10% were obese [6]. These trends have since escalated, with the latest WHO/COSI data for 2022 showing that 30.2% of children aged 8 to 9 years were overweight, of whom 14.2% were obese. This prevalence was higher among girls (16.7% vs. 15.4% for boys) and in urban areas (15.7% vs. 11.8% for rural areas) [5]. Additionally, the most recent Global Nutrition Report for 2019 reported the prevalence of overweight in children and adolescents aged 5 to 19 years as being 28.7% (higher in boys than girls, 33.8% vs. 23.7%), and the prevalence of obesity as being 9.7% (higher in boys than girls, 12.2% vs. 7.2%) [4].

In this context, childhood overnutrition represents an alarming health challenge, as it impacts current health and increases the risk of developing chronic diseases in adulthood. The Albanian government has prioritized addressing overweight and obesity through initiatives such as the “Monitoring of obesity every 3 years in children aged 6–9.9 years as part of the European Childhood Obesity Surveillance Initiative”. Additionally, NCD prevention and control are priorities included in the “Health Strategy, Albania 2021–2030”, in the “Action Plan on NCDs, Albania 2021–2030”, and the “Action Plan on Health Promotion, Albania 2022–2030” [5]. However, these frameworks lack clearly defined targets for reducing overweight and obesity among youths [7].

Nutrition plays a pivotal role in the prevention and control of overweight, obesity and NCDs. The nutrition transition is characterized by changes in eating habits, including an increased consumption of highly processed foods rich in fats, sugars, and salt [8]. Although multiple factors contribute to this shift, the adoption of modern lifestyles, particularly among youths, is a driver [9]. Numerous epidemiological studies highlight the protective effects of the Mediterranean Diet (MD) in preventing NCDs [10,11]. Key components of the MD include olive oil as the primary cooking fat, a high consumption of plant-based foods, and a moderate intake of meat. Despite its benefits in comparison with other types of diets, adherence to the MD has declined globally in recent decades as a result of the nutrition transition. Albania, whose traditional cuisine aligns with the MD, lacks comprehensive studies on this topic [12]. Specifically, data on the nutritional status and dietary patterns of youths are scarce, with the last ‘2022 Report of the European Commission’ highlighting the urgent need to raise awareness of dietary risks [13]. It is also crucial to address disparities in health and nutrition in Albania, which are often linked to age, gender, socio-economic status, and geographic location [14]. The socio-economic transformation that has taken place during the transitional stage of the Albanian economy is still jeopardized in the country, exacerbating disparities, particularly in rural areas where 37% of the population resides [15]. Gender inequality remains a significant issue [16], especially in rural and remote areas, where traditional patriarchal systems persist [14].

To bridge these knowledge gaps, we conducted a cross-sectional study examining the anthropometric status and dietary habits of Albanian school-aged children and adolescents living in rural and semi-urban areas. This study aims to draft a representation of the nutrition profile for a sample of youths in the era of nutrition transition in Albania and to identify potential factors associated with poor dietary habits and malnutrition.

## 2. Materials and Methods

### 2.1. Study Design and Population

This cross-sectional study was conducted between October and November 2024 in various rural and semi-urban areas in Albania: Lezha (north), Rrëshen (north), Elbasan (central), Shelcan (central), and Milzë (central). A convenience sample of students aged 10–18 years (grades VI–XII) attending secondary schools was selected. Specifically, two upper secondary schools in Lezha and Rrëshen (with eight and six classes, respectively), one lower secondary school in Elbasan (five classes), one lower secondary school in Shelcan (four classes), and one lower secondary school in Mlizë (three classes) were included in the study.

### 2.2. Ethical Considerations

Before data collection, initial contact was made with school authorities in each city. The school directors were briefed on the study’s objectives and methodology, and a copy of the questionnaire was provided in advance. Teachers were briefed with sufficient details regarding the study (procedures including anthropometric and dietary habits assessment), and information related to the anonymity of the study was explained. The school passed the information to the children’s parents. Formal approval from ethical review committees was not requested. However, the study adhered to the “Helsinki World Medical Association Declaration” (1975)—Ethical Principles For Medical Research Involving Human subjects”. Passive consent was obtained from the parents through teachers. On the scheduled evaluation day, students had been pre-informed about the questionnaire and anthropometric measurements. Data collection commenced only after participants provided oral informed consent. Participation in the study was voluntary. No pupils absent on the evaluation day were assessed afterwards.

### 2.3. Data Collection

Interviews were conducted face-to-face by trained teams, each of which underwent a practical session on fieldwork procedures conducted by members of the Albanian Society of Nutrition Science (ASNS). Team composition varied by location: in Lezha, the team included a PhD student, two medical students from the Catholic University “Our Lady of Good Councel” of Tirane, and two nurses; in Rrëshen, a PhD student and two medical students participated; in Elbasan, a PhD student and one nurse were involved; in Shelcan e Mlizë a PhD student and a teacher carried out the interviews.

### 2.4. Questionnaire and Measurements

Data were collected using a printed questionnaire divided into three sections: a first part with socio-demographic information, a second part with anthropometric measurements, and a third part with dietary habits. Each participant was assigned an ID number to ensure privacy and confidentiality. The questionnaire was administered in the Albanian language.

Sociodemographic characteristics included variables such as gender, age, place of residence, and religion.

Anthropometric measurements included weight and height. They were taken on-site using portable scales and stadiometers. Weight measurements were accurate to ±100 g, and height measurements to ±1 cm. Body mass index (BMI) was calculated according to age- and sex-specific international WHO standards [17]. BMI-for-age Z-scores (BAZ) and height-for-age Z scores (HAZ) were calculated using WHO Anthro Software (Version 3.2.2, January 2011, WHO, Geneva, Switzerland) [18]. Nutritional status was classified as follows [17]:

- Overweight: BAZ > +1 SD;
- Obesity: BAZ > +2 SD;
- Underweight: BAZ < −2 SD;
- Stunting (chronic malnutrition): HAZ < −2 SD.

To evaluate adherence to the Mediterranean Diet (MD), the Mediterranean Diet Quality Index in Children and Adolescents (KIDMED) was used. KIDMED is the most widely spread simple and straightforward validated questionnaire to evaluate the quality of the diet for children and adolescents [19]. The index consists of 16 questions reflecting core MD principles. Each question has two possible answers, assigned a score of 1 or −1, and provided with a total score ranging from 0 to 12. By summing the results, adherence to the MD is defined using three levels of quality, as follows:

- ≤3 very low;
- 4–7 intermediate;
- ≥8 high or optimal.

The validated Italian version of the KIDMED questionnaire [20] was used and translated into Albanian. The instrument was translated by two independent bilingual Albanian–Italian academicians. Back translation was carried out to ensure accuracy.

### 2.5. Statistical Analysis

A database containing anonymized data was generated by inputting information for the analysis. Data were analyzed using the Statistical Package for Social Sciences (SPSS version 26.0, IBM, Somers, NY, USA). Continuous variables were reported as means with standard deviations (SDs), while categorical variables were presented as numbers and percentages. Differences in means for continuous variables were assessed using the Student t-test, and the Pearson’s chi-squared test was used to compare categorical variables. ANOVA with Bonferroni post hoc testing was used to evaluate differences among age groups. Odds ratios (ORs) with a 95% confidence interval (95% CI) were calculated to identify factors influencing dietary patterns. A multivariate logistic regression model AOR (95%CI) was created to identify key drivers for poor adherence to the MD (KIDMED score ≤ 3). As there were multiple independent variables, a stepwise forward regression approach was used. Statistical significance was set at *p*-value ≤ 0.05.

## 3. Results

Of the 426 children included in the study, 201 (47.2%) were female. Only two children refused to participate, and four additional questionnaires were excluded due to incomplete information. Sociodemographic information is presented in Table 1. The overall mean age was 15.3 years ± 1.9 SD, with a median age of 15.7 years (range 10–18 years). Children under 13 years accounted for 16.0% of the sample (54.4% of them were females), 42.0% were aged 13–16 years (29.6% female), and 42.0% were aged above 16 years (62.0% female). Regarding religion, 57.3% of children were Catholics, 33.1% were Muslims, 9.4% were Orthodox (9.4%), and 0.2% were Jehovah’s Witness. Slightly more than half of the sample (55.6%) resided in rural areas (Rrëshen: n.152, Mlize: n.45, and Shelcan: n.40), especially females; 44.4% of the pupils interviewed resided in semi-urban areas (Elbasan: n.73, and Lezha: n.116).

Table 2 presents the anthropometric characteristics for the overall sample and by age groups, with ANOVA tests used to detect differences in mean values. For BAZ, no significant differences were found between the genders across any age group. In contrast, HAZ revealed statistically significant gender differences in children aged 13–16 years (mean HAZ 0.2 ± 0.9 SD for females vs. 0.6 ± 1.1 SD for males, *p* = 0.007). No significant differences in HAZ were found between genders for children under 13 years or above 16 years. Additionally, no differences in anthropometric parameters were detected by area of residence across age groups.

When considering nutritional status classification, more than 20% of the sample exceeded the ideal weight (being either overweight or obese). The prevalence of overnutrition tended to decrease with age, being highest among children younger than 13 years. No statistically significant differences in the prevalence of malnutrition were observed by area of residence.

Table 3 summarizes the KIDMED results. The overall mean KIDMED score was 4.6 ± 2.5 SD (range −2 to 12 points), indicating an intermediate level of adherence to the MD. Statistically significant differences were found between females and males (4.1 ± 2.4 SD vs. 5.1 ± 2.4 SD, *p* < 0.001). When examining geographic differences, females living in rural areas had lower KIDMED scores than their male counterparts (3.9 ± 2.4 SD vs. 4.9 ± 2.5 SD, *p* = 0.003), while no significant gender differences were noted among urban youths.

Among children aged 13–16 years, significant gender-based differences in the KIDMED scores were observed (mean value 4.1 ± 2.4 SD for females vs. 5.2 ± 2.5 SD for males, *p* = 0.01). Similarly, gender differences were detected in children aged above 16 years (mean value 3.8 ± 2.5 SD for females vs. 4.8 ± 2.4 SD for males, *p* = 0.008). No significant gender differences were found among children under 13 years old.

When considering areas of residence, the only difference in the total KIDMED score was detected in the 13–16 years age group (mean value 4.1 ± 2.4 SD for rural vs. 5.1 ± 2.5 SD for urban, *p* = 0.012).

Regarding MD adherence, 34.3% of the overall sample demonstrated poor dietary habits, 53.3% showed moderate adherence, and 12.4% exhibited high-quality dietary habits. Poor adherence increased with age, while a peak in high dietary quality was observed in the 13–16 age group. These trends were consistent across genders, although differences among age groups were not statistically significant.

**Figure 1:**
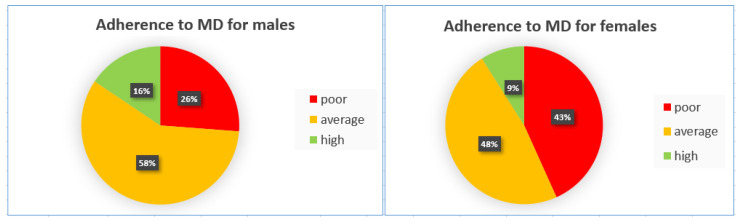
Comparisons of adherence to MD between genders.

When stratified by gender (Figure 1), a statistically significant difference in MD adherence was noted, with females exhibiting poorer dietary habits across all KIDMED classification categories (*p* < 0.001).

**Figure 2:**
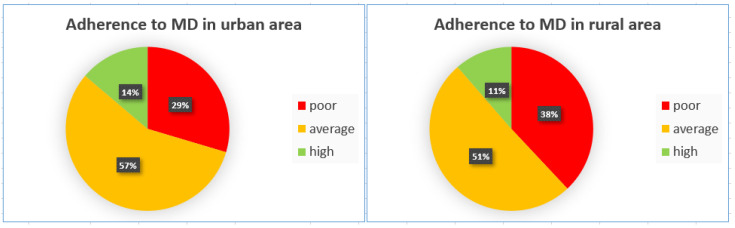
Comparisons of adherence to MD between areas of residence.

When stratifying by residence (Figure 2), children living in urban areas had a better adherence to the MD than their rural counterparts, although these differences were not statistically significant.

**Figure 3:**
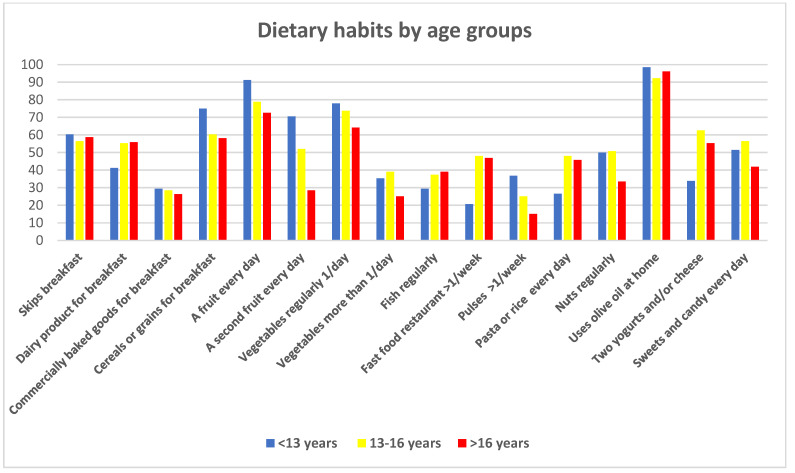
Dietary habits by age groups.

Figure 3 depicts dietary habits by age group. Daily fruit consumption (both one and multiple portions) decreased with age (*p* = 0.007 and *p* < 0.001, respectively). Similar trends were observed for pulses (*p* = 0.001), nuts (*p* = 0.002), and vegetables (*p* = 0.05 for one portion; *p* < 0.05 for the second portion), with a peak in consumption for children aged 13–16 years.

The oldest children reported higher frequencies of eating at fast food and at restaurants (*p* < 0.001). The youngest children consumed sweets daily more frequently (*p* = 0.02). The middle age group consumed two portions of yogurt or cheese daily more frequently than their counterparts (*p* < 0.001). Other differences in dietary consumption by age were not statistically significant.

**Figure 4:**
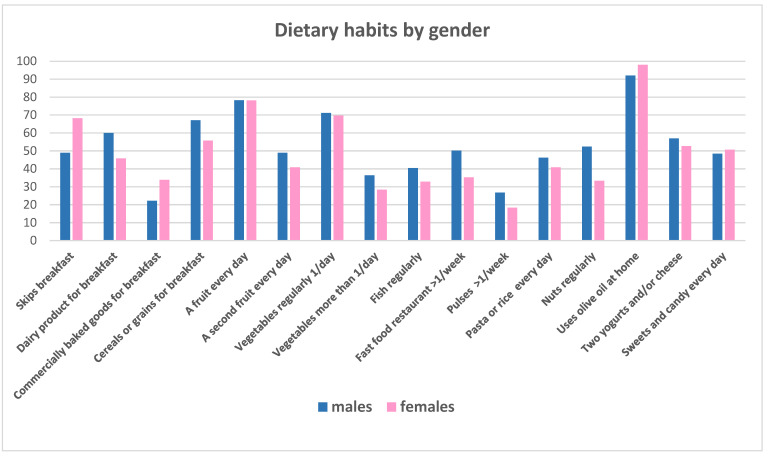
Dietary habits for total cohort and by gender.

When analyzing dietary habits by gender (Figure 4), males exhibited better outcomes: males consumed more pulses (*p* < 0.05), cereals for breakfast (*p* = 0.01), and nuts (*p* < 0.001). Conversely, females were less likely to eat at fast food or restaurants (*p* = 0.001), were more likely to use oil olive at home (*p* = 0.004), and consumed fewer commercially baked products for breakfast (*p* = 0.005). When considering poor practices, an increased risk of skipping breakfast was found among girls (OR 2.2; CI 1.5–3.3; *p* < 0.001), as well as lower consumption of dairy products for breakfast (OR 1.7; CI 1.2–2.6; *p* = 0.002).

**Figure 5:**
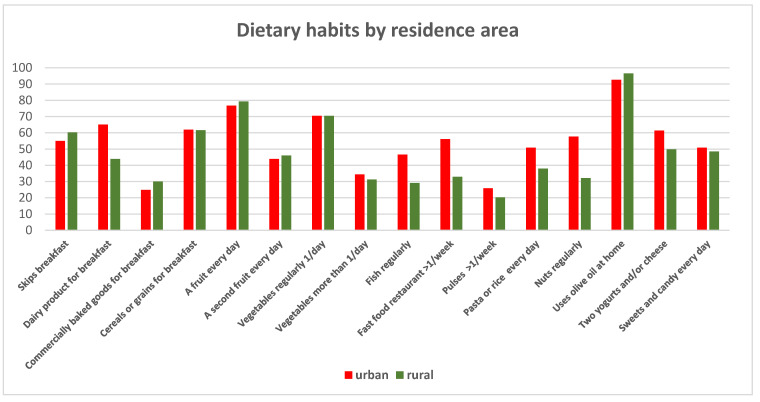
Dietary habits for total cohort and by residence area.

Figure 5 examines dietary habits by area of residence. Children in rural areas consumed fish less frequently (*p* < 0.001), while urban children were more likely to eat at fast food and restaurants (OR 2.6, CI 1.7–3.9; *p* < 0.001). Nuts were most frequently consumed in urban areas, along with dairy products for breakfast (*p* < 0.001). Urban children also consumed two daily portions of dairy products more often (*p* = 0.01).

Other differences in eating behaviors by residence were not statistically significant.

Table 4 highlights the main factors associated with poor adherence to the MD, calculated as KIDMED score ≤ 3. After adjusting for model covariates, multivariate analysis revealed that being female was the strongest predictor of poor MD adherence (AOR 2.08; 95% CI: 1.34–3.22; *p* = 0.001).

## 4. Discussion

The nutritional transition has led to a generalized trend of adopting unhealthy eating behaviors in Western countries. However, the underlying determinants of these shifts are complex and vary significantly across regions, and within individual countries [21]. In the Balkans, research on the nutritional status and quality of the dietary habits of children and adolescents in the era of the nutritional transition remains limited.

This preliminary study assessed the nutritional status and dietary behaviors of schoolchildren (aged 10–18 years) living in rural and semi-urban areas of Albania. Regarding malnutrition, over 20% of the sample was classified as overweight or obese. The prevalence of overnutrition was lower than national estimates [5], but slightly higher than findings in school-aged children living in rural areas of Albania (12% overweight, 6.2% obese) [22]. Among the youngest group, our results surpassed those reported in a Greek study of primary school students aged 10–12 years (21.7% overweight, 5.0% obese) [23]. Unlike the studies by Kanellopoulou et al. and by Hyska et al., our findings showed no significant gender differences in anthropometric parameters [22,23]. Additionally, in contrast to Hyska and colleagues, we found that the prevalence of obesity was similar across different areas of residence, while the prevalence of underweight was consistent with their findings (3.2%) [22]. In the present study, it was observed that overnutrition decreased with age, despite unhealthy eating habits increasing with age. Although this may initially seem contradictory, these findings can potentially be explained by the natural physiological growth process, where food intake requirements generally increase. This increased need may compensate for unhealthy behaviors, especially during adolescence, when autonomy in making food choices grows. As adolescents gain more control over their diet, their preferences may become misaligned with traditional family eating patterns, yet the physiological demand for nutrition might still balance out the effects of unhealthy habits [24].

The dietary patterns observed in this study highlighted a suboptimal adherence to the Mediterranean Diet among Albanian youths, aligning with national data indicating unhealthy eating habits in children [25]. At present, few studies on the same topic have been conducted in the country to compare our results with. The poor MD adherence in our study nearly doubled the rate reported by Llanaj and Hanley-Cook (34% vs. 19%), while, consequently, the rate of good adherence was lower (13% vs. 18%) [26]. However, it is important to note that the latter study was focused on university students (aged 18–24 years) in Tirana, the Albania’s largest urban center. To the best of our knowledge, the most recent study evaluating the nutritional status and dietary habits of Albanian schoolchildren, including those living in rural settings, was a nationwide survey conducted in 2017–2018 [22]. Similar to Hyska et al.’s study, we found that skipping breakfast was prevalent, with our findings being higher. According to their findings, girls were more prone to skip breakfast even in our study. As a matter of fact, gender differences in food intake and selection first appear in adolescence, with girls experiencing more food-related conflicts and greater dissatisfaction with their body shape than boys [27]. Further investigations are needed to clarify to what extent the differences between genders were conditioned by physiological, psychological, and socio-cultural factors [28].

Our results revealed a significantly lower consumption of dairy products at breakfast among younger children, girls, and those living in rural areas. Limited data exist for direct comparison, but these trends may stem from poor breakfast habits. Additionally, national reports have indicated declining dairy production in rural areas between 2020 and 2022 [29].

A significant disparity in fish consumption between geographic locations was found; we can potentially assume that the lower rate for rural settings might be due to the coexistence of traditional food habits, economic factors, and geographic distance from coastal regions.

When comparing findings from other Mediterranean regions, our results aligned with a multicenter study conducted across Croatia, Greece, Israel, Italy, Macedonia, and Serbia, where MD adherence was intermediate in all countries except Serbia [30]. Results on dietary quality tending to decline from childhood to adolescence, as for total and in both sexes—with the lowest MD adherence being observed in children aged >16 years—mirror a study conducted in Italy on a vulnerable population of children (KIDMED mean score ≈ 4), although the latter involved a younger cohort (6–12 years) [31]. Contrary to Kanellopoulou and colleagues, no significant differences in KIDMED scores were found between normal-weight and overweight/obese children in our study [23]. Additionally, unhealthy dietary habits (such as skipping breakfast, frequent fast food and restaurant, and excessive sweets intake) were not associated with nutritional status, diverging from the findings of Tambalis and colleagues [32].

Significant differences in dietary habits were observed between semi-urban and rural areas, with rural children reporting poorer adherence to the MD, especially those aged 13–16 years. Prior studies have shown that, globally, rural settings face greater nutritional imbalances due to socio-cultural, economic, and lifestyle factors [33]. In the Western Balkans, rural areas experience hindrances due to different factors including food insecurity [34]. Household food insecurity is a recognized factor contributing to poor nutrition among many Albanian children [22].

The study by Harris-Fry and colleagues highlighted how food imbalances are exacerbated in regions with pronounced gender inequities [33]. At present, in Albania, a formal legal system aligned with the European Union coexists with traditional patriarchal societal norms, which particularly disfavor women in rural areas. In a patriarchal context, family eating practices are often shaped by prevailing social norms, making it challenging to promote an equitable distribution of food within the household. This can lead to inequality, which may disproportionately affect young adolescent girls [35]. When patriarchy is combined with poverty, gender inequality is exacerbated [36]. Therefore, the gender-based inequalities in nutrition habits found in the present study might be interpreted in light of the entrenched patriarchal system, especially in remote regions.

### Limitations

As a preliminary investigation, this study has several limitations. First, it is a cross-sectional study so our findings should be interpreted with caution given the study design (i.e., lack of a control group, and follow-up investigation). Second, the convenience sample was not representative of all regions of Albania, particularly urban areas and major cities. Third, the statistical analysis did not account for potential confounding factors such as physical activity and access to social media, which may have impacted our findings. Additionally, recall bias may exist in a self-report investigation, but this challenge cannot be fully avoided; we can assume that the amount of information bias is comparable to that of other similar epidemiological studies since the self-reported questionnaire has been extensively validated in prior research among schoolchildren [19]. Given these limitations, we acknowledge that our study’s findings cannot be generalized to all Albanian schoolchildren or the broader population. Further investigations are needed to confirm this preliminary investigation.

## 5. Conclusions

The unhealthy eating habits and high rate of overweight/obesity among Albanian youths reflect the ongoing nutrition transition in the country. This study highlights the need to prioritize healthy nutrition lifestyles, like the Mediterranean Diet, as a cornerstone for ensuring the long-term wellbeing of Albanian younger generations. These preliminary results hold significant public health relevance in a region with high mortality rates due to cardiovascular diseases. Future nutritional interventions and policies should take into account geographic, cultural, and social factors—including gender equality—that can influence dietary patterns, particularly in the rural areas of Albania.

## Figures and Tables

**Table 1:** Socio-demographic characteristics of the overall sample.

Variables	Values
Sex	*n* (%)
Female	201 (47.2)
Males	225 (52.8)
Age, years	Mean ± SD
	15.3 ± 1.9
Age groups	*n* (%)
<13	68 (16.0)
13–16	179 (42.0)
>16	179 (42.0)
Age range	10–18 years
School grade	*n* (%)
VI	40 (9.4)
VII	47 (11.0)
VIII	39 (9.2)
IX	32 (7.5)
X	116 (27.2)
XI	82 (19.2)
XII	70 (16.4)
Religion	*n* (%)
Christian Catholics	244 (57.3)
Christian Orthodox	40 (9.4)
Muslims	141 (33.1)
Jehovah’s Witness	1 (0.2)
Area of residence	*n* (%)
Semi-urban	189 (44.4)
Rural	237 (55.6)

**Table 2:** Anthropometric characteristics of the overall sample and by age groups.

Variables	Total (n.426)	<13 Years (n.68)	13–16 Years (n.179)	>16 Years (n.179)	*p*-Value (ANOVA Test)
Weight (kg)	59.1 ± 12.9	49.2 ± 13.5	58.3 ± 12.1	63.5 ± 11.2	All statistically significant < 0.001
Height (cm)	167.4 ± 10.8	154.1 ± 8.9	168.7 ± 9.5	171.1 ± 8.7	All statistically significant < 0.001 except 13–16 vs. >16 = 0.045
Body Mass Index (BMI)	20.9 ± 3.5	20.5 ± 4.5	20.4 ± 3.6	21.6 ± 2.7	All NS except 13–16 vs. >16 = 0.004
BMI for Age Z-score (BAZ)	0.1 ± 1.1	0.7 ± 1.3	0.1 ± 1.1	−0.02 ± 1.1	All NS
Height for Age Z-score (HAZ)	0.5 ± 1.0	0.6 ± 1.1	0.5 ± 1.1	0.6 ± 0.9	All statistically significant < 0.001 except 13–16 vs. >16 NS
Malnutrition	*n* (%)	*n* (%)	*n* (%)	*n* (%)	*p*-value ^a^
Overweight	68 (15.9)	18 (26.5)	24 (13.4)	26 (14.5)	<0.001
Obesity	25 (5.9)	10 (14.7)	10 (5.6)	5 (2.8)	=0.002
Underweight	16 (3.8)	2 (2.9)	6 (3.4)	8 (4.5)	NS
Stunting	3 (0.7)	1 (1.5)	2 (1.1)	0 (0)	NS

^a^ χ2 test.

**Table 3:** KIDMED score for total and by age group.

Variables	Total (n.426)	<13 Years (n.68)	13–16 Years (n.179)	>16 Years (n.179)	*p* Values (ANOVA Test)
KIDMED score	4.6 ± 2.5	5.0 ± 2.2	4.9 ± 2.5	4.1 ± 2.5	<13 vs. >16 = 0.04 13–16 vs. >16 = 0.02
Adherence to MD	*n* (%)	*n* (%)	*n* (%)	*n* (%)	*p*-value ^a^
Poor	146 (34.3)	17 (25.0)	58 (32.4)	71 (39.7)	NS
Average	227 (53.3)	43 (63.2)	94 (52.5)	90 (50.3)	
High	53 (12.4)	8 (11.8)	27 (15.1)	18 (10.1)	

^a^ χ2 test.

**Table 4:** Univariate and multivariate logistic regression analysis for factors associated with low adherence to MD.

	Univariate Analysis		Multivariate Analysis	
Variable	OR (95%CI)	*p*-Value	AOR (95%CI)	*p*-Value
Gender (female)	2.15 (1.42–3.22) *	<0.001	2.08 (1.34–3.22) *	0.001
Age	0.92 (0.83–1.03)	0.156	0.84 (0.84–1.04)	0.266
Residence area (rural)	1.45 (0.97–2.19)	0.044	1.05 (0.67–1.67)	0.823

* Statistically significant results.

## Data Availability

The data presented in this study are available on request from the corresponding author due to privacy and ethical reasons.

## Abbreviations

The following abbreviations are used in this manuscript:

MD	Mediterranean Diet
KIDMED	Mediterranean Diet Quality Index in Children and Adolescents
ORs	Odds Ratios
AOR	Adjusted odds ratio
CI	Confidence interval
NCDs	Non-communicable diseases
CVDs	Cardiovascular diseases
GBD	Global Burden of Disease
WHO	World Health Organizations
COSI	Childhood Obesity Surveillance Initiative
ASNS	Albanian Society of Nutrition Science
BMI	Body mass index
BAZ	BMI for age Z-scores
HAZ	Height-for-age Z scores
SPSS	Statistical Package for Social Sciences
SD	Standard Deviations
